# Light-Fueled Synchronization of Two Coupled Liquid Crystal Elastomer Self-Oscillators

**DOI:** 10.3390/polym15132886

**Published:** 2023-06-29

**Authors:** Kai Li, Biao Zhang, Quanbao Cheng, Yuntong Dai, Yong Yu

**Affiliations:** Department of Civil Engineering, Anhui Jianzhu University, Hefei 230601, China

**Keywords:** self-excited motion, liquid crystal elastomer, synchronization, domain of attraction

## Abstract

The synchronization and group behaviors of self-excited coupled oscillators are common in nature and deserve to be explored, for self-excited motions have the advantages of actively collecting energy from the environment, being autonomous, making equipment portable, and so on. Based on light-powered self-excited oscillators composed of liquid crystal elastomer (LCE) bars, the synchronization of two self-excited coupled oscillators is theoretically studied. Numerical calculations show that self-excited oscillations of the system have two synchronization modes, in-phase mode and anti-phase mode, which are mainly determined by their interaction. The time histories of various quantities are calculated to elucidate the mechanism of self-excited oscillation and synchronization. For strong interactions, the system always develops into in-phase synchronization mode, while for weak interaction, the system will evolve into anti-phase synchronization mode. Furthermore, the effects of initial conditions, contraction coefficient, light intensity, and damping coefficient on the two synchronization modes of the self-excited oscillation are investigated extensively. The initial condition generally does not affect the synchronization mode and its amplitude. The amplitude of self-oscillation always increases with increasing contraction coefficient, gravitational acceleration, and light intensity, while it decreases with the increasing damping coefficient. This work will deepen people’s understanding of the synchronization behaviors of self-excited coupled oscillators, and the theoretical framework could be extended to scenarios involving large-scale synchronization of the systems with numerous interacting oscillators.

## 1. Introduction

Self-excited oscillation is a phenomenon of periodic state change within a system under constant external stimulation [1,2,3,4,5,6,7]. Because of its unique advantages, self-oscillation has wide application prospects in many fields, such as energy harvesting [8,9], signal sensors [10], soft robotics [11,12,13,14,15], mechano-logistic devices [16,17], biomimetic designs [18], and so on. First, self-oscillation can directly harvest energy from the constant external environment in order to maintain its own periodic motion, which is similar to the effect of biological active feeding. Second, the periodic motion of self-oscillation does not need periodic external stimulation, only constant external stimulation. This feature greatly reduces the requirements of system motion control and does not need a complex controller. Third, the above characteristics of no controller and battery greatly reduce the complexity of the active machine and make it more portable, which is expected to achieve high power [19,20,21]. In many conventional and active material systems, different self-excited oscillations have recently been constructed [22,23,24].

In nonconservative oscillation, the energy loss of self-excited oscillation caused by system damping needs external energy input and energy compensation [25,26,27,28,29]. Recently, based on different stimuli-responsive materials and structures, different feedback mechanisms have been proposed to realize energy compensation [22,23,24,30]. The stimuli-responsive materials for self-excited oscillation systems include hydrogels [31,32], ionic gels [33], liquid crystal elastomer (LCE) [34], and so on. Meanwhile, different feedback mechanisms include coupling of chemical reactions and large deformation [25,26,35], self-shading effect [22,23,36], multi-process coupling of droplet evaporation, and plate bending [27]. Self-excited oscillations come from the nonlinear coupling of multiple processes of the system. Among the stimuli-responsive materials, LCE has the advantages of fast response, recoverable deformation, and low noise [17,37,38,39,40]. LCE is a polymer network structure formed by cross-linking liquid crystal monomer molecules. Under the stimulation of external fields, such as light, heat, electricity, and magnetism, the liquid crystal monomer molecules will rotate or undergo phase transition and change the configuration to induce macroscopic deformation [38,41,42,43,44]. A great deal of experimental and theoretical work has been carried out on self-excited oscillations based on LCE [22,23,24,45,46,47,48,49,50,51,52,53,54,55].

Although a great deal of work has been carried out on single self-excited oscillators, the interaction and collective motion of multiple self-excited oscillators need to be further explored. Synchronization and collective motion are ubiquitous in nature, such as the circadian rhythm and cardiac pacemaker cells [56,57,58,59,60]. The first work on synchronization can be traced back to Huygens’ observations in 1665 on the synchronization of coupled pendulums [61]. He observed that two identical clocks oscillate synchronously with two pendulums in opposite directions. Recent studies have confirmed that the synchronization between two pendulums results from the coupling caused by small mechanical vibrations that propagate in the wooden structure connecting the clocks [62]. Furthermore, the synchronous movement of a large number of metronomes with more degrees of freedom on a free moving base is also demonstrated experimentally [63]. Recently, based on light-responsive LCE, Ghislaine et al. experimentally studied the synchronized oscillations of thin plastic actuators fueled by light and found two kinds of synchronous oscillation phenomena of in-phase and anti-phase in steady state [64,65]. Their numerical simulations qualitatively explain the origin of the synchronized motion and found that the motion can be tuned by the mechanical properties of the coupling joint.

At present, there are few studies on the interaction and group phenomenon of self-excited coupled oscillators based on active materials [64], and the synchronization mechanism and its behavior need to be further explored. In this paper, based on a self-excited oscillator made of photoresponsive LCE previously proposed by us [66], we investigate the synchronous behavior of two identical self-excited oscillators powered by steady illumination. This paper is as follows. First, based on dynamic LCE model [67], the dynamic governing equation for two identical self-excited oscillators under steady illumination is formulated in Section 2. Second, two kinds of synchronization mode of the self-excited oscillations are presented in Section 3. In Section 4 and 5, the detailed self-excited mechanism of in-phase and anti-phase modes are elucidated, respectively. Meanwhile, the influences of initial conditions and spring constant on the synchronization mode, amplitude, and period of the self-excited oscillations are investigated. Finally, the conclusion is given in Section 6.

## 2. Model and Formulation

In this section, a theoretical model is proposed for two identical LCE oscillators connected by torsional springs under steady illumination. It mainly includes the dynamic model of two LCE oscillators, evolution law of number fraction in the two LCE oscillators, non-dimensionalization and solution method of the differential governing equations with variable coefficients.

### 2.1. Dynamic Model of the two LCE Oscillators

**Figure 1:**
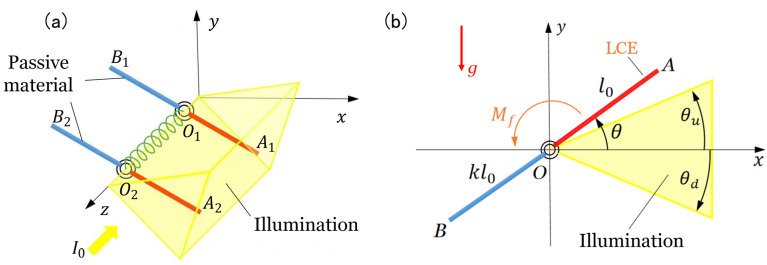
Schematics of dynamic model of two identical LCE oscillators connected by a torsion spring under steady illumination. The LCE oscillators are made up of a light-responsive LCE bar and a passive material bar. The region from $\theta_{d}$ to $\theta_{u}$ is steadily illuminated. (**a**) Oblique view. (**b**) Side view.

Figure 1a sketches the dynamic model of two identical LCE oscillators connected by a torsion spring under steady illumination. $O_{1}A_{1}$ and $O_{2}A_{2}$ are light-responsive LCE bars, while $O_{1}B_{1}$ and $O_{2}B_{2}$ are passive material bars. The four bars have the same mass m. Bars $A_{1}O_{1}B_{1}$ and $A_{2}O_{2}B_{2}$ can rotate around the z axis. The original length of $O_{1}A_{1}$ and $O_{2}A_{2}$ is $l_{0}$ and the length of $O_{1}B_{1}$ and $O_{2}B_{2}$ is $kl_{0}$, with k being the length ratio. The connecting torsion spring between the two bars allows them to apply corresponding torque to one another, depending on the relative angle difference between the two bars. The illumination zone is denoted by the upper edge angle $\theta_{u}$ and the lower edge angle $\theta_{d}$, as shown in Figure 1b. The positions of $O_{1}A_{1}$ and $O_{2}A_{2}$ are denoted by $\theta_{1}$ and $\theta_{2}$, respectively. The initial angles of $O_{1}A_{1}$ and $O_{2}A_{2}$ are $\theta_{1}^{0}$ and $\theta_{2}^{0}$, respectively. The initial angular velocity is of $O_{1}A_{1}$ and $O_{2}A_{2}$ are ${\dot{\theta }}_{1}^{0}$ and ${\dot{\theta }}_{2}^{0}$, respectively. Under light illumination, bar $O_{1}A_{1}$ and bar $O_{2}A_{2}$ can oscillate, because the light-driven deformation periodically changes the center of gravity and reverses the resultant moment of the system due to the self-shadowing effect. In the following, we will investigate the synchronization of the two self-excited oscillations.

According to the theorem of moment of momentum, the differential equations for the dynamics of the two LCE bars rotating around the fixed z axis are

(1) $$\frac{d\Psi_{1}}{dt}=M_{z1},\;\frac{d\Psi_{2}}{dt}=M_{z2},$$

where the angular momenta of $A_{1}O_{1}B_{1}$ and $A_{2}O_{2}B_{2}$ are, respectively,

(2) $$\Psi_{1}=J_{z1}\left(t\right)\frac{d\theta_{1}\left(t\right)}{dt},\;\Psi_{2}=J_{z2}\left(t\right)\frac{d\theta_{2}\left(t\right)}{dt},$$

where the moments of inertia of $A_{1}O_{1}B_{1}$ and $A_{2}O_{2}B_{2}$ about z axis are, respectively,

(3) $$J_{z1}=J_{A1}+J_{B1},\;J_{z2}=J_{A2}+J_{B2},$$

where $J_{A1}=\frac{1}{3}ml_{1}^{2}$ is the moment of inertia of $O_{1}A_{1}$ about z axis, $J_{B1}=\frac{1}{3}mk^{2}l_{0}{}^{2}$ is the moment of inertia of $O_{1}B_{1}$ about z axis, $J_{A2}=\frac{1}{3}ml_{2}^{2}$ is the moment of inertia of $O_{2}A_{2}$ about z axis, and $J_{B2}=\frac{1}{3}mk^{2}l_{0}{}^{2}$ is the moment of inertia of $O_{2}B_{2}$ about z axis. It is worth noting that the length depends on the light-driven contraction strain. Then, the length $l_{1}$ of the $O_{1}A_{1}$ and the length $l_{2}$ of the $O_{2}A_{2}$ can be expressed as

(4) $$l_{1}=\left[1+\varepsilon_{1}(t)\right]l_{0},\;l_{2}=\left[1+\varepsilon_{2}(t)\right]l_{0},$$

where $\varepsilon_{1}(t)$ and $\varepsilon_{2}(t)$ are the light-driven contraction strains of $O_{1}A_{1}$ and $O_{2}A_{2}$, respectively. For simplicity, we assume that the light-driven contraction strain of the material is proportional to the number fraction φ(t),

(5) $$\varepsilon_{1}(t)=-C_{0}\varphi_{1}(t),\;\varepsilon_{2}(t)=-C_{0}\varphi_{2}(t),$$

where $C_{0}$ is the contraction coefficient. The number fraction φ(t) is given in Section 2.2.

In Equation (1), $M_{z1}$ and $M_{z2}$ are the resultant moments of all external forces of bar $A_{1}O_{1}B_{1}$ and bar $A_{2}O_{2}B_{2}$ to *z*-axis, respectively,

(6) $$M_{Z1}=M_{D1}-M_{f1}+M_{K},\;M_{Z2}=M_{D2}-M_{f2}+M_{K},$$

where the driving moments of $A_{1}O_{1}B_{1}$ and $A_{2}O_{2}B_{2}$ can easily be calculated as

(7) $$M_{D1}=\frac{1}{2}mg\left(kl_{0}\cos\theta_{1}-l_{1}\cos\theta_{1}\right),\;M_{D2}=\frac{1}{2}mg\left(kl_{0}\cos\theta_{2}-l_{2}\cos\theta_{2}\right),$$

where g is the gravitational acceleration.

The damping force is assumed to be proportional to the velocity, and then the damping moments of $A_{1}O_{1}B_{1}$ and $A_{2}O_{2}B_{2}$ can be easily calculated as

(8) $$M_{f1}=\frac{1}{3}\beta \left(l_{1}^{3}+k^{3}l_{0}^{3}\right)\frac{d\theta_{1}}{dt},\;M_{f2}=\frac{1}{3}\beta \left(l_{2}^{3}+k^{3}l_{0}^{3}\right)\frac{d\theta_{2}}{dt},$$

where β is the damping coefficient, $\frac{d\theta_{1}}{dt}={\dot{\theta }}_{1}$ is the angular velocity of $A_{1}O_{1}B_{1}$ and $\frac{d\theta_{2}}{dt}={\dot{\theta }}_{2}$ is the angular velocity of $A_{2}O_{2}B_{2}$.

The moment exerted by the torsion spring on the two bars is assumed to be linear with the angle difference,

(9) $$M_{K}=\alpha \left(\theta_{1}-\theta_{2}\right),$$

where α is the spring coefficient of the torsion spring.

### 2.2. Evolution Law of Number Fraction in the Two LCE Oscillators

In order to calculate the light-driven contraction strain and the lengths of the LCE bars, we need to obtain the number fractions in the LCE bars. According to the research of Yu et al., the *trans*-to-*cis* isomerization of LCE can be induced by UV or laser with wavelength less than 400 nm [68]. Generally, the *cis*-to-*trans* isomerization driven by UV light and the thermal *trans*-to-*cis* excitation can be neglected. Therefore, the number fraction of *cis*-isomers depends on the thermal excitation from *trans* to *cis*, the thermally driven relaxation from *cis* to *trans* and the light-driven *trans*-to-*cis* isomerization. Then, the number fraction of bent *cis*-isomers in LCE can be governed by the following equation [67,68],

(10) $$\frac{\partial \varphi }{\partial t}=\eta_{0}I_{0}(1-\varphi )-\frac{\varphi }{T_{0}},$$

where $T_{0}$ is the thermal relaxation time of *cis* state to *trans* state, $I_{0}$ is the light intensity, and $\eta_{0}$ is the light absorption constant. By solving Equation (10), the number fraction of *cis*-isomers can be expressed as:

(11) $$\varphi (t)=\frac{\eta_{0}T_{0}I_{0}}{\eta_{0}T_{0}I_{0}+1}+(\varphi_{0}-\frac{\eta_{0}T_{0}I_{0}}{\eta_{0}T_{0}I_{0}+1})\exp\left[-\frac{t}{T_{0}}(\eta_{0}T_{0}I_{0}+1)\right],$$

where $\varphi_{0}$ is the number fraction of *cis*-isomers at t=0. In the light zone, for initially zero number fraction of *cis*-isomers, i.e., $\varphi_{0}=0$, Equation (11) can be simplified as

(12) $$\varphi (t)=\frac{\eta_{0}T_{0}I_{0}}{\eta_{0}T_{0}I_{0}+1}\left\{1-\exp\left[-\frac{t}{T_{0}}\left(1+\eta_{0}T_{0}I_{0}\right)\right]\right\}.$$

In the dark zone, namely $I_{0}=0$, $\varphi_{0}$ can be set as the maximum value of φ(t) in Equation (12), namely, $\varphi_{0}=\frac{\eta_{0}T_{0}I_{0}}{\eta_{0}T_{0}I_{0}+1}$, and Equation (11) can be simplified as:

(13) $$\varphi \left(t\right)=\frac{\eta_{0}T_{0}I_{0}}{\eta_{0}T_{0}I_{0}+1}\exp\left(-\frac{t}{T_{0}}\right).$$

### 2.3. Nondimensionalization

By defining the following dimensionless quantities: $\bar{t}=t/T_{0}$, $\bar{I}=\eta_{0}T_{0}I_{0}$,  $\bar{g}={\left(T_{0}/\sqrt{l_{0}/g}\right)}^{2}$, $\bar{\beta }=2\beta l_{0}T_{0}/m$, $\bar{\alpha }=\alpha T_{0}^{2}/\left(ml_{0}^{2}\right)$, $\bar{\varphi }=\varphi \left(\eta_{0}T_{0}I_{0}+1\right)/\eta_{0}T_{0}I_{0}$,  ${\bar{M}}_{K}=M_{K}T_{0}^{2}/\left(ml_{0}^{2}\right)$, ${\bar{M}}_{D1}=2M_{D1}T_{0}^{2}/\left(ml_{0}^{2}\right)$ and ${\bar{M}}_{D2}=2M_{D2}T_{0}^{2}/\left(ml_{0}^{2}\right)$, in the light zone, Equation (12) is rewritten as:

(14) $$\bar{\varphi }=1-\exp\left[-\bar{t}(\bar{I}+1)\right],$$

in the dark zone, Equation (13) becomes:

(15) $$\bar{\varphi }=\exp(-\bar{t}).$$

A combination of Equations (1)–(4), (14) and (15) can yield, in the light zone:

(16) $$\frac{d^{2}\theta_{1}}{d{\bar{t}}_{1}^{2}}=\frac{4C_{0}\bar{I}\left(1+\varepsilon_{1}\right)\exp\left[-{\bar{t}}_{1}\left(\bar{I}+1\right)\right]-\bar{\beta }\left[{\left(1+\varepsilon_{1}\right)}^{3}+k^{3}\right]}{2{\left(1+\varepsilon_{1}\right)}^{2}+2k^{2}}\frac{d\theta_{1}}{d{\bar{t}}_{1}}+\frac{3\bar{g}\left(k-\varepsilon_{1}-1\right)\cos\theta_{1}-6\bar{\alpha }\left(\theta_{1}-\theta_{2}\right)}{2{\left(1+\varepsilon_{1}\right)}^{2}+2k^{2}},$$

(17) $$\frac{d^{2}\theta_{2}}{d{\bar{t}}_{2}^{2}}=\frac{4C_{0}\bar{I}\left(1+\varepsilon_{2}\right)\exp\left[-{\bar{t}}_{2}\left(\bar{I}+1\right)\right]-\bar{\beta }\left[{\left(1+\varepsilon_{2}\right)}^{3}+k^{3}\right]}{2{\left(1+\varepsilon_{2}\right)}^{2}+2k^{2}}\frac{d\theta_{2}}{d{\bar{t}}_{2}}+\frac{3\bar{g}\left(k-\varepsilon_{2}-1\right)\cos\theta_{2}+6\bar{\alpha }\left(\theta_{1}-\theta_{2}\right)}{2{\left(1+\varepsilon_{2}\right)}^{2}+2k^{2}},$$

where $\varepsilon_{1}=-C_{0}\bar{I}\left\{1-\exp\left[-{\bar{t}}_{1}\left(\bar{I}+1\right)\right]\right\}/\left(\bar{I}+1\right)$ and $\varepsilon_{2}=-C_{0}\bar{I}\left\{1-\exp\left[-{\bar{t}}_{2}\left(\bar{I}+1\right)\right]\right\}/\left(\bar{I}+1\right)$, in the dark zone:

(18) $$\frac{d^{2}\theta_{1}}{d{\bar{t}}_{1}^{2}}=\frac{-4C_{0}\bar{I}\left(1+\varepsilon_{1}\right)\exp\left(-{\bar{t}}_{1}\right)/\left(\bar{I}+1\right)-\bar{\beta }\left[{\left(1+\varepsilon_{1}\right)}^{3}+k^{3}\right]}{2{\left(1+\varepsilon_{1}\right)}^{2}+2k^{2}}\frac{d\theta_{1}}{d{\bar{t}}_{1}}+\frac{3\bar{g}\left(k-\varepsilon_{1}-1\right)\cos\theta_{1}-6\bar{\alpha }\left(\theta_{1}-\theta_{2}\right)}{2{\left(1+\varepsilon_{1}\right)}^{2}+2k^{2}},$$

(19) $$\frac{d^{2}\theta_{2}}{d{\bar{t}}_{2}^{2}}=\frac{-4C_{0}\bar{I}\left(1+\varepsilon_{2}\right)\exp\left(-{\bar{t}}_{2}\right)/\left(\bar{I}+1\right)-\bar{\beta }\left[{\left(1+\varepsilon_{2}\right)}^{3}+k^{3}\right]}{2{\left(1+\varepsilon_{2}\right)}^{2}+2k^{2}}\frac{d\theta_{2}}{d{\bar{t}}_{2}}+\frac{3\bar{g}\left(k-\varepsilon_{2}-1\right)\cos\theta_{2}+6\bar{\alpha }\left(\theta_{1}-\theta_{2}\right)}{2{\left(1+\varepsilon_{2}\right)}^{2}+2k^{2}},$$

where $\varepsilon_{1}=-C_{0}\bar{I}\exp\left(-{\bar{t}}_{1}\right)/\left(\bar{I}+1\right)$ and $\varepsilon_{2}=-C_{0}\bar{I}\exp\left(-{\bar{t}}_{2}\right)/\left(\bar{I}+1\right)$.

In summary, Equations (16)–(19) depict the dynamics of two coupled LCE oscillators rotating in the light zone and in the dark zone, respectively. It is noted that the evolution of the cis number fraction in two LCE oscillators differs in two zones, and the variation of the light-driven contraction strain and the lengths of the LCE bars, which rely on the cis content, is process-related.

### 2.4. Solution Method

Equations (16)–(19) are ordinary differential equations with variable coefficients, and there are no analytic solutions. Hereon, the classical fourth-order Runge–Kutta method is utilized to solve ordinary differential equations by MATLAB software. Extensively used Runge–Kutta explicit iterative formulas are detailed as follows,

(20) $$y_{n+1}=y_{n}+\frac{H}{6}\left(k_{1}+2k_{2}+2k_{3}+k_{4}\right),$$

where H is the time step, and $k_{i}$ (i = 1 to 4) are listed as below,

(21) $$\left\{\begin{array}{c} k_{1}=f\left({\bar{t}}_{n},y_{n}\right)\\ k_{2}=f\left({\bar{t}}_{n}+\frac{H}{2},y_{n}+\frac{H}{2}k_{1}\right)\\ k_{3}=f\left({\bar{t}}_{n}+\frac{H}{2},y_{n}+\frac{H}{2}k_{2}\right)\\ k_{4}=f\left({\bar{t}}_{n}+H,y_{n}+Hk_{3}\right) \end{array}\right..$$

Each of Equations (16)–(19), second-order ordinary differential equations (ODEs), needs to be transformed into two first-order ODEs in accordance with the numerical rules in Matlab. Herein, $f\left({\bar{t}}_{n},y_{n}\right)$ in Equation (21) takes a two-dimensional vector form by zoning as below, in the light zone:

(22) $$\left[\begin{array}{l} \;\frac{d\theta_{1}}{d{\bar{t}}_{1}}\\ \frac{4C_{0}\bar{I}\left(1+\varepsilon_{1}\right)\exp\left[-{\bar{t}}_{1}\left(\bar{I}+1\right)\right]-\bar{\beta }\left[{\left(1+\varepsilon_{1}\right)}^{3}+k^{3}\right]}{2{\left(1+\varepsilon_{1}\right)}^{2}+2k^{2}}\frac{d\theta_{1}}{d{\bar{t}}_{1}}+\frac{3\bar{g}\left(k-\varepsilon_{1}-1\right)\cos\theta_{1}-6\bar{\alpha }\left(\theta_{1}-\theta_{2}\right)}{2{\left(1+\varepsilon_{1}\right)}^{2}+2k^{2}} \end{array}\right],$$

(23) $$\left[\begin{array}{l} \;\frac{d\theta_{2}}{d{\bar{t}}_{2}}\\ \frac{4C_{0}\bar{I}\left(1+\varepsilon_{2}\right)\exp\left[-{\bar{t}}_{2}\left(\bar{I}+1\right)\right]-\bar{\beta }\left[{\left(1+\varepsilon_{2}\right)}^{3}+k^{3}\right]}{2{\left(1+\varepsilon_{2}\right)}^{2}+2k^{2}}\frac{d\theta_{2}}{d{\bar{t}}_{2}}+\frac{3\bar{g}\left(k-\varepsilon_{2}-1\right)\cos\theta_{2}+6\bar{\alpha }\left(\theta_{1}-\theta_{2}\right)}{2{\left(1+\varepsilon_{2}\right)}^{2}+2k^{2}} \end{array}\right],$$

in the dark zone:

(24) $$\left[\begin{array}{l} \;\frac{d\theta_{1}}{d{\bar{t}}_{1}}\\ \frac{-4C_{0}\bar{I}\left(1+\varepsilon_{1}\right)\exp\left(-{\bar{t}}_{1}\right)/\left(\bar{I}+1\right)-\bar{\beta }\left[{\left(1+\varepsilon_{1}\right)}^{3}+k^{3}\right]}{2{\left(1+\varepsilon_{1}\right)}^{2}+2k^{2}}\frac{d\theta_{1}}{d{\bar{t}}_{1}}+\frac{3\bar{g}\left(k-\varepsilon_{1}-1\right)\cos\theta_{1}-6\bar{\alpha }\left(\theta_{1}-\theta_{2}\right)}{2{\left(1+\varepsilon_{1}\right)}^{2}+2k^{2}} \end{array}\right],$$

(25) $$\left[\begin{array}{l} \;\frac{d\theta_{2}}{d{\bar{t}}_{2}}\\ \frac{-4C_{0}\bar{I}\left(1+\varepsilon_{2}\right)\exp\left(-{\bar{t}}_{2}\right)/\left(\bar{I}+1\right)-\bar{\beta }\left[{\left(1+\varepsilon_{2}\right)}^{3}+k^{3}\right]}{2{\left(1+\varepsilon_{2}\right)}^{2}+2k^{2}}\frac{d\theta_{2}}{d{\bar{t}}_{2}}+\frac{3\bar{g}\left(k-\varepsilon_{2}-1\right)\cos\theta_{2}+6\bar{\alpha }\left(\theta_{1}-\theta_{2}\right)}{2{\left(1+\varepsilon_{2}\right)}^{2}+2k^{2}} \end{array}\right].$$

Equations (22)–(25) in combination with the initial conditions $\theta_{1}^{0}$, ${\dot{\theta }}_{1}^{0}$, and $\theta_{2}^{0}$, ${\dot{\theta }}_{2}^{0}$ constitute the closed system of dynamic equations describing the two coupled LCE oscillators connected by a torsion spring under steady illumination. The self-sustained motion of the LCE oscillators, i.e., the variation of angle and angular velocity with time, can be obtained by iteration.

## 3. Two Synchronization Modes

In this section, based on solving the governing Equations (22)–(25), we present two synchronization modes, namely in-phase mode and anti-phase mode. In order to study the synchronization mode of two LCE oscillators under steady illumination, it is necessary to determine the typical values of the dimensionless parameters. The typical material properties and geometric parameters obtained from the existing tests [37,69] are shown in Table 1, and the corresponding dimensionless parameters are shown in Table 2. In the following, these values of parameters are used to study the synchronization modes of two LCE oscillators under steady illumination.

**Figure 2:**
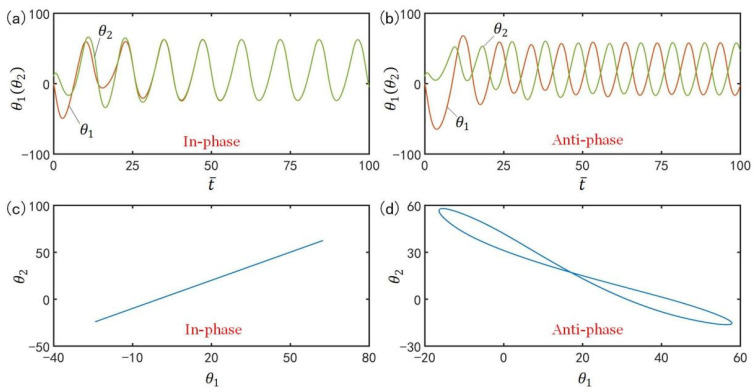
Two types of synchronization modes of the light-powered self-excited LCE oscillators. The parameters are $\bar{I}=0.33$, $C_{0}=0.4$, $\bar{g}=9.8$, k=9.2, $\bar{\beta }=0.9$, ${\dot{\theta }}_{1}^{0}=-0.33$, ${\dot{\theta }}_{2}^{0}=0.33$, $\theta_{u}=45{}^{\circ}$, $\theta_{d}=-80{}^{\circ}$, $\theta_{1}^{0}=0^{\circ }$ and $\theta_{2}^{0}=10^{\circ }$. (**a**,**b**) correspond to two typical synchronization modes: in-phase mode for $\bar{\alpha }=0.1$ and anti-phase mode for $\bar{\alpha }=0.03$. (**c**,**d**) are the respective domains of attraction of the two typical synchronization modes.

Figure 2 shows two typical synchronization modes of self-excited motion of LCE oscillators: in-phase mode and anti-phase mode. In the computation, we fix $\bar{I}=0.33$, $C_{0}=0.4$, $\bar{g}=9.8$, k=9.2, $\bar{\beta }=0.9$, ${\dot{\theta }}_{1}^{0}=-0.33$, ${\dot{\theta }}_{2}^{0}=0.33$, $\theta_{u}=45^{\circ }$, $\theta_{d}=-80^{\circ }$, $\theta_{1}^{0}=0^{\circ }$ and $\theta_{2}^{0}=10^{\circ }$. The time history curve and domain of attraction of the in-phase mode for $\bar{\alpha }=0.1$ are given in Figure 2a,c, respectively. The results show that the two LCE bars oscillate in in-phase mode. Figure 2b,d present the time history curve and domain of attraction of the anti-phase mode for $\bar{\alpha }=0.03$, respectively. The results show that the two LCE bars oscillate in anti-phase mode. Through careful calculation, it is found that there exists a critical spring constant ${\bar{\alpha }}_{\mathrm{crit}}=0.075$ for the two synchronization modes. The two LCE oscillators will oscillate in in-phase mode for $\bar{\alpha }>{\bar{\alpha }}_{\mathrm{crit}}$, while they oscillate in anti-phase mode for $\bar{\alpha }<{\bar{\alpha }}_{\mathrm{crit}}$. In the following, we will discuss the two synchronization modes in turn.

## 4. In-Phase Synchronization Mode

In the above mechanical model of two coupled LCE oscillators, there are five dimensionless parameters, including $\bar{I}$, $\bar{g}$, $\bar{\alpha }$, $\bar{\beta }$, and $C_{0}$. In this section, the mechanism of self-oscillation in in-phase mode, and the influences of dimensionless parameters on the in-phase synchronous mode are studied in details.

### 4.1. Mechanisms of the Self-Excited Oscillation in In-Phase Mode

**Figure 3:**
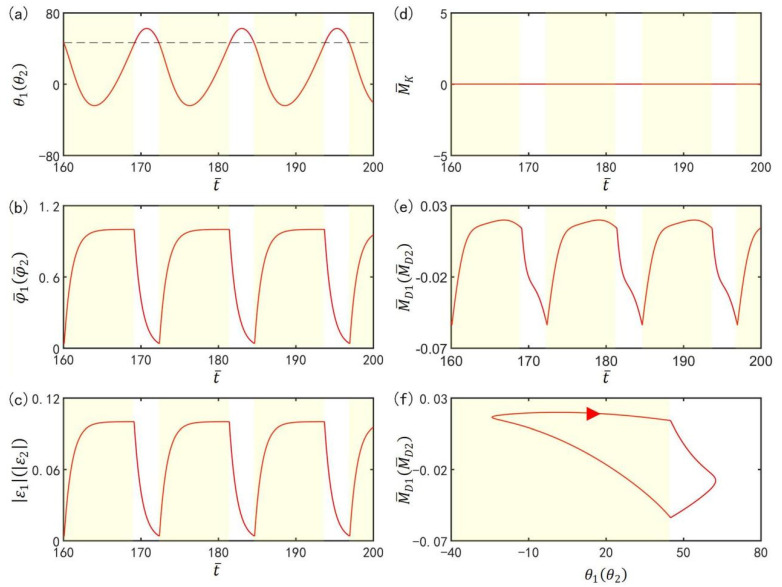
Mechanism of the self-excited oscillation in in-phase mode. The parameters are $\bar{I}=0.33$, $C_{0}=0.4$, $\bar{g}=9.8$, k=9.2, $\bar{\beta }=0.9$, $\bar{\alpha }=0.1$, ${\dot{\theta }}_{1}^{0}=-0.3$, ${\dot{\theta }}_{2}^{0}=0.3$, $\theta_{u}=45^{\circ }$, $\theta_{d}=-80^{\circ }$, $\theta_{1}^{0}=0^{\circ }$, and $\theta_{2}^{0}=10^{\circ }$. (**a**) Time histories of $\theta_{1}$ or $\theta_{2}$. (**b**) Time histories of the number fractions of *cis*-isomers in the two LCE bars. (**c**) Time histories of contraction strains. (**d**) Time history of moment of the torsion spring (**e**) Time histories of the driving moments. (**f**) The relationship between the driving moments and $\theta_{1}$ or $\theta_{2}$.

To investigate the mechanism of the self-excited oscillation in in-phase mode of the two LCE oscillators under steady illumination, Figure 3 presents time-histories of some key physical quantities of the in-phase mode in Figure 2b,d. Figure 3a plots the time histories of $\theta_{1}$ or $\theta_{2}$, which shows that the two LCE bars oscillate periodically in in-phase mode. Figure 3b plots time histories of the number fractions of *cis*-isomers in the two LCE bars. The number fractions of *cis*-isomers increase in the light zone, while they decrease in the dark zone. Therefore, the contraction strains in the two LCE bars increase in the light zone while decreasing in the dark zone, as shown in Figure 3c. In Figure 3d, the moment of the torsion spring on the two bars is zero, because the angle difference between the two bars in in-phase mode is zero. In Figure 3e, the driving moments of the two oscillators also change periodically in in-phase mode. Figure 3f delineates the dependence of the driving moment on $\theta_{1}$ or $\theta_{2}$. In Figure 3f, the area surrounded by the closed curve represents the net work performed by the steady illumination during one cycle of the self-excited oscillation, which compensates for the energy dissipation caused by the damping to maintain the oscillation of the LCE oscillators.

### 4.2. Effect of the Spring Constant on the In-Phase Mode

**Figure 4:**
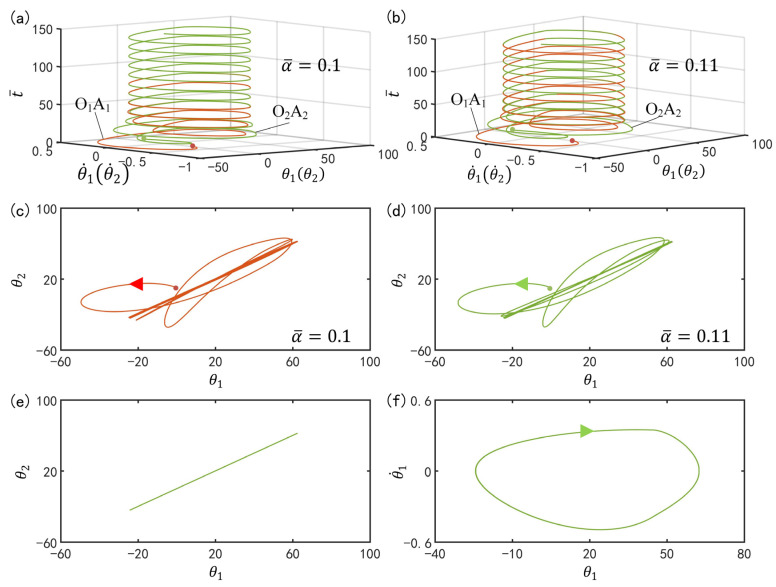
Effect of spring constant on the in-phase mode. The parameters are $\bar{I}=0.33$, $C_{0}=0.4$, $\bar{g}=9.8$, k=9.2, $\bar{\beta }=0.9$, ${\dot{\theta }}_{1}^{0}=-0.33$, ${\dot{\theta }}_{2}^{0}=0.33$, $\theta_{u}=45^{\circ }$, $\theta_{d}=-80^{\circ }$, $\theta_{1}^{0}=0^{\circ }$ and $\theta_{2}^{0}=10^{\circ }$. (**a**) Time history for $\bar{\alpha }=0.1$; (**b**) Time history for $\bar{\alpha }=0.11$; (**c**) Phase diagrams for $\bar{\alpha }=0.1$; (**d**) Phase diagrams for $\bar{\alpha }=0.11$; (**e**) Domain of attraction of $\theta_{1}$ and $\theta_{2}$ for different spring constants; (**f**) Limit cycles of $\theta_{1}$ and ${\dot{\theta }}_{1}$ for different spring constants.

Figure 4a,b plot the time histories of the two oscillators for different spring constants. In this computation, we set $\bar{I}=0.33$, $C_{0}=0.4$, $\bar{g}=9.8$, k=9.2, $\bar{\beta }=0.9$, ${\dot{\theta }}_{1}^{0}=-0.33$, ${\dot{\theta }}_{2}^{0}=0.33$, $\theta_{u}=45^{\circ }$, $\theta_{d}=-80^{\circ }$, $\theta_{1}^{0}=0^{\circ }$ and $\theta_{2}^{0}=10^{\circ }$. In Figure 4a,b, for the two different spring constants, the two curves of the two LCE oscillators coincide after a period of time, which means that the two bars oscillate synchronously in in-phase mode. Figure 4c,d plot the phase diagrams for the two spring constants, in which $\theta_{1}$ and $\theta_{2}$ evolve from the initial disorder into a final attraction domain. Figure 4e further plots the domains of attraction of $\theta_{1}$ and $\theta_{2}$ for different spring constants. The calculation shows that the two domains of attraction are identical. The results show that the spring constant has no effect on the in-phase mode.

**Figure 5:**
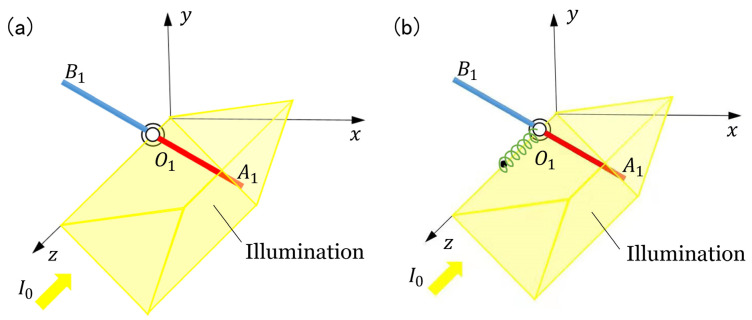
Equivalent systems of (**a**) in-phase synchronization mode, and (**b**) anti-phase synchronization mode. In in-phase mode, the system is equivalent to the single oscillator. In anti-phase mode, the system is equivalent to the single oscillator with half-length torsion spring.

Furthermore, Figure 4f describes the limit cycles of $\theta_{1}$ and ${\dot{\theta }}_{1}$ for different spring constants. Similarly, the two limit cycles are also the same. This implies that the spring constant has no effect on the amplitude and frequency of the LCE oscillators. More calculations show that the influence of the spring constant is the same for $\bar{\alpha }>{\bar{\alpha }}_{\mathrm{crit}}=0.075$. This is because for $\bar{\alpha }>{\bar{\alpha }}_{\mathrm{crit}}$, the LCE oscillators are in in-phase mode, and both the angle difference between the two bars and the moment of the spring are zero. Therefore, the spring constant has no effect on its amplitude and frequency. In in-phase mode, the system is equivalent to the single oscillator, as shown in Figure 5a.

### 4.3. Effect of the Initial Conditions on the In-Phase Mode

**Figure 6:**
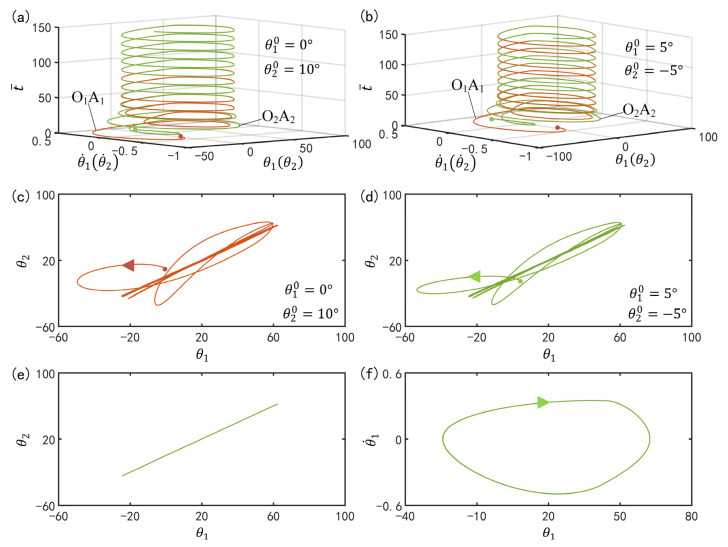
Effect of initial conditions on the in-phase mode. The parameters are $\bar{I}=0.33$, $C_{0}=0.4$, $\bar{g}=9.8$, k=9.2, $\bar{\beta }=0.9$, $\bar{\alpha }=0.1$, ${\dot{\theta }}_{1}^{0}=-0.33$, ${\dot{\theta }}_{2}^{0}=0.33$, $\theta_{u}=45{}^{\circ}$ and $\theta_{d}=-80{}^{\circ}$. (**a**) Time history for $\theta_{1}^{0}=0{}^{\circ}$ and $\theta_{2}^{0}=10{}^{\circ}$; (**b**) Time history for $\theta_{1}^{0}=5{}^{\circ}$ and $\theta_{2}^{0}=-5{}^{\circ}$; (**c**) Phase diagrams for $\theta_{1}^{0}=0{}^{\circ}$ and $\theta_{2}^{0}=10{}^{\circ}$; (**d**) Phase diagrams for $\theta_{1}^{0}=5{}^{\circ}$ and $\theta_{2}^{0}=-5{}^{\circ}$; (**e**) Domain of attraction of $\theta_{1}$ and $\theta_{2}$ for different initial conditions; (**f**) Limit cycles of $\theta_{1}$ and ${\dot{\theta }}_{1}$ for different initial conditions.

Figure 6a,b plot the time–histories for two different initial conditions. In the computation, we set $\bar{I}=0.33$, $C_{0}=0.4$, $\bar{g}=9.8$, k=9.2, $\bar{\beta }=0.9$, $\bar{\alpha }=0.1$, ${\dot{\theta }}_{1}^{0}=-0.33$, ${\dot{\theta }}_{2}^{0}=0.33$, $\theta_{u}=45{}^{\circ}$ and $\theta_{d}=-80{}^{\circ}$. In Figure 6a,b, for the two different initial conditions, the two curves of the two LCE oscillators coincide after a period of time, which means that the two bars oscillate synchronously in in-phase mode. Figure 6c,d plot the phase diagrams for the two initial conditions, in which $\theta_{1}$ and $\theta_{2}$ evolve from the initial disorder into a final attraction domain. Figure 6e further plots the domains of attraction of $\theta_{1}$ and $\theta_{2}$ for different initial conditions. The calculation shows that the two domains of attraction are the same. The results show that the initial condition has no effect on the synchronous mode.

Furthermore, Figure 6f describes the limit cycles of $\theta_{1}$ and ${\dot{\theta }}_{1}$ for different initial conditions. Similarly, the two limit cycles are also identical. This implies that the amplitude and frequency of the LCE oscillators are independent on the initial condition, which is further validated by more calculations. It is noted that the effect of initial condition on amplitude and frequency is similar to that of the single LCE oscillator [66].

### 4.4. Effect of the Contraction Coefficient on the In-Phase Mode

**Figure 7:**
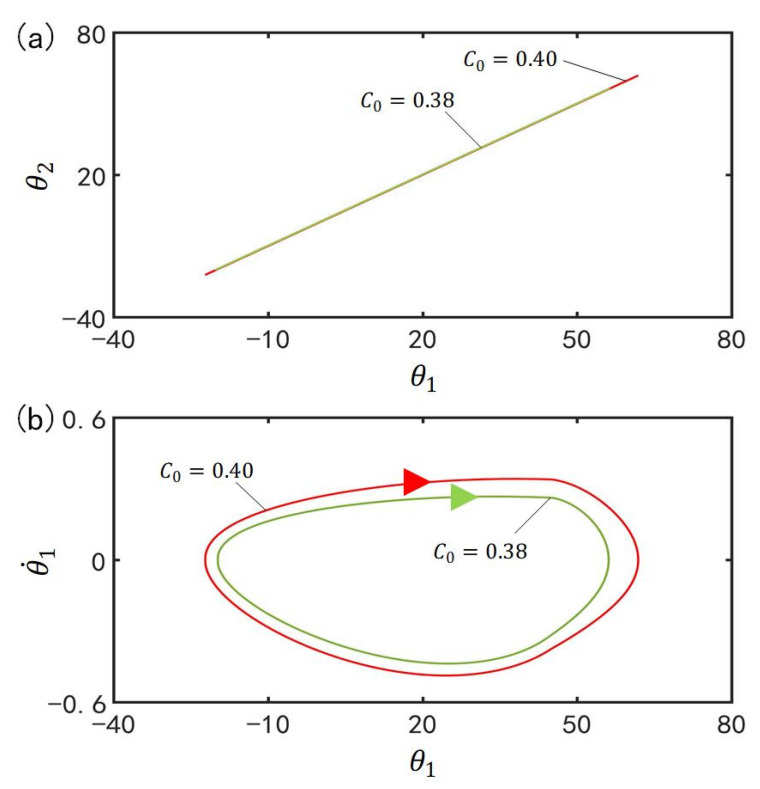
Effect of contraction coefficient $C_{0}$ on the in-phase mode for $\bar{I}=0.33$, $\bar{\alpha }=0.1$, $\bar{g}=9.8$, k=9.2, $\bar{\beta }=0.9$, ${\dot{\theta }}_{1}^{0}=-0.33$, ${\dot{\theta }}_{2}^{0}=0.33$, $\theta_{u}=45{}^{\circ}$, $\theta_{d}=-80{}^{\circ}$, $\theta_{1}^{0}=0{}^{\circ}$, and $\theta_{2}^{0}=10{}^{\circ}$. (**a**) Domain of attraction of $\theta_{1}$ and $\theta_{2}$ for different contraction coefficients; (**b**) Limit cycles of $\theta_{1}$ and ${\dot{\theta }}_{1}$ for different contraction coefficients.

Figure 7 plots the effect of contraction coefficient $C_{0}$ on the in-phase mode for $\bar{I}=0.33$, $\bar{\alpha }=0.1$, $\bar{g}=9.8$, k=9.2, $\bar{\beta }=0.9$, ${\dot{\theta }}_{1}^{0}=-0.33$, ${\dot{\theta }}_{2}^{0}=0.33$, $\theta_{u}=45{}^{\circ}$, $\theta_{d}=-80{}^{\circ}$, $\theta_{1}^{0}=0{}^{\circ}$, and $\theta_{2}^{0}=10{}^{\circ}$. Figure 7a plots the domains of attraction of $\theta_{1}$ and $\theta_{2}$ for different contraction coefficients. Furthermore, Figure 7b describes the limit cycles of $\theta_{1}$ and ${\dot{\theta }}_{1}$ for different contraction coefficients. It can be seen that its amplitude increases with the increase of the contraction coefficient. This is because with the increase of contraction coefficient $C_{0}$, the light-driven contraction of the LCE bar increases and then the center of gravity of the LCE bar changes greatly, which results in the augmentation of the driving moment and the amplitude of the LCE bar.

### 4.5. Effect of the Light Intensity on the In-Phase Mode

**Figure 8:**
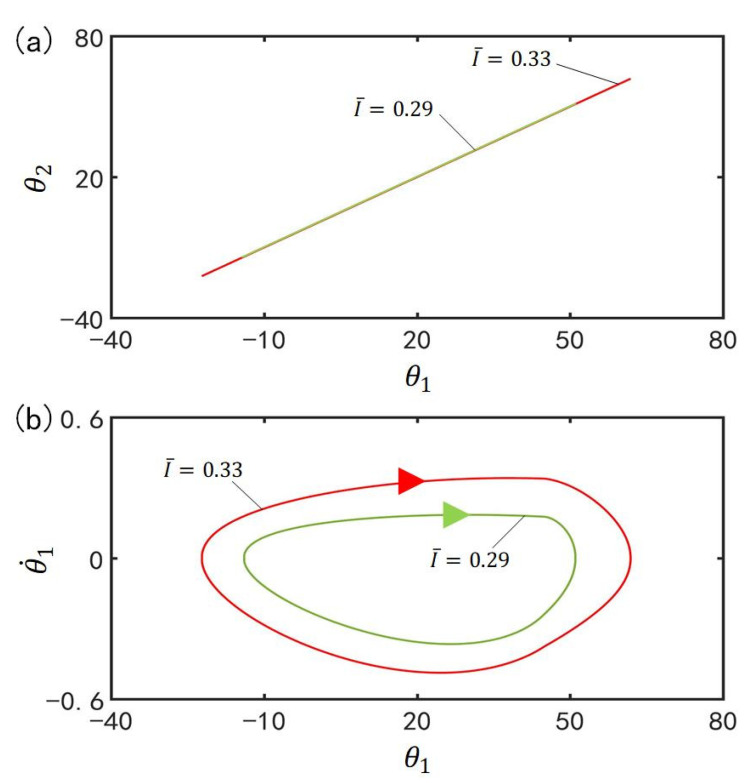
Effect of light intensity $\bar{I}$ on the in-phase mode for $C_{0}=0.4$, $\bar{\alpha }=0.1$, $\bar{g}=9.8$, k=9.2, $\bar{\beta }=0.9$, ${\dot{\theta }}_{1}^{0}=-0.33$, ${\dot{\theta }}_{2}^{0}=0.33$, $\theta_{u}=45{}^{\circ}$, $\theta_{d}=-80{}^{\circ}$, $\theta_{1}^{0}=0{}^{\circ}$, and $\theta_{2}^{0}=10{}^{\circ}$. (**a**) Domain of attraction of $\theta_{1}$ and $\theta_{2}$ for different light intensities; (**b**) Limit cycles of $\theta_{1}$ and ${\dot{\theta }}_{1}$ for different light intensities.

Figure 8 plots the effect of light intensity $\bar{I}$ on the in-phase mode for $C_{0}=0.4$, $\bar{\alpha }=0.1$, $\bar{g}=9.8$, k=9.2, $\bar{\beta }=0.9$, ${\dot{\theta }}_{1}^{0}=-0.33$, ${\dot{\theta }}_{2}^{0}=0.33$, $\theta_{u}=45{}^{\circ}$, $\theta_{d}=-80{}^{\circ}$, $\theta_{1}^{0}=0{}^{\circ}$, and $\theta_{2}^{0}=10{}^{\circ}$. Figure 8a plots the domains of attraction of $\theta_{1}$ and $\theta_{2}$ for different light intensities. Furthermore, Figure 8b describes the limit cycles of $\theta_{1}$ and ${\dot{\theta }}_{1}$ for different light intensities. Similar to the effect of the contraction coefficient, its amplitude increase with the increase of the light intensity. This is because with the rise of light intensity $\bar{I}$, the light-driven deformation of the LCE bar increases and then the center of gravity of the LCE bar changes greatly, which leads to the augmentation of the driving moment and the amplitude of the self-excited oscillation.

### 4.6. Effect of the Damping Coefficient on the In-Phase Mode

**Figure 9:**
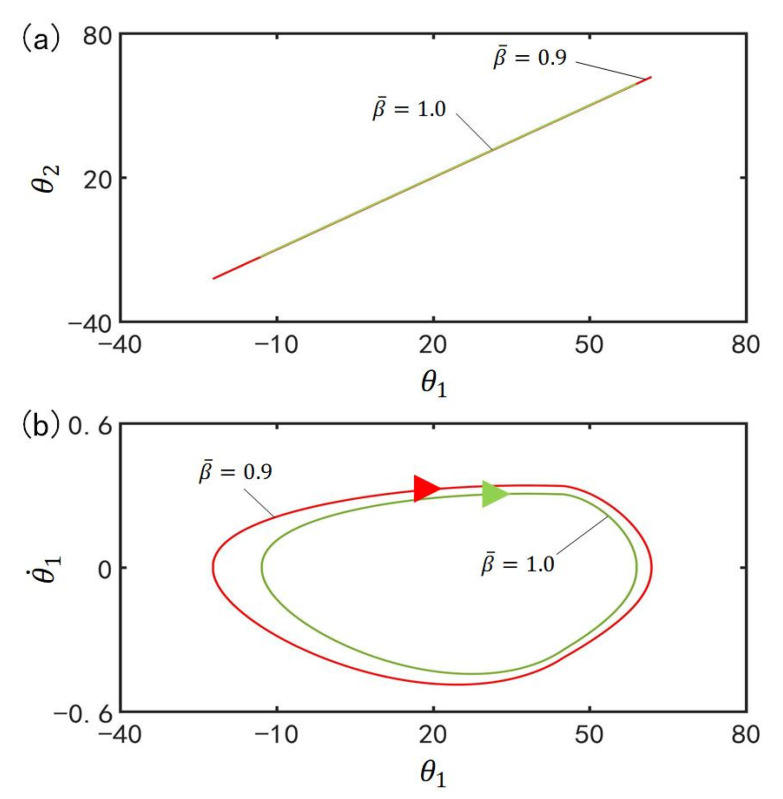
Effect of damping coefficient $\bar{\beta }$ on the in-phase mode for $\bar{I}=0.33$, $C_{0}=0.4$, $\bar{g}=9.8$, k=9.2, $\bar{\alpha }=0.1$, ${\dot{\theta }}_{1}^{0}=-0.33$, ${\dot{\theta }}_{2}^{0}=0.33$, $\theta_{u}=45{}^{\circ}$, $\theta_{d}=-80{}^{\circ}$, $\theta_{1}^{0}=0{}^{\circ}$, and $\theta_{2}^{0}=10{}^{\circ}$. (**a**) Domain of attraction of $\theta_{1}$ and $\theta_{2}$ for different damping coefficients; (**b**) Limit cycles of $\theta_{1}$ and ${\dot{\theta }}_{1}$ for different damping coefficients.

Figure 9 plots the effect of damping coefficient $\bar{\beta }$ on the in-phase mode for $\bar{I}=0.33$, $C_{0}=0.4$, $\bar{g}=9.8$, k=9.2, $\bar{\alpha }=0.1$, ${\dot{\theta }}_{1}^{0}=-0.33$, ${\dot{\theta }}_{2}^{0}=0.33$, $\theta_{u}=45{}^{\circ}$, $\theta_{d}=-80{}^{\circ}$, $\theta_{1}^{0}=0{}^{\circ}$, and $\theta_{2}^{0}=10{}^{\circ}$. Figure 9a plots the domains of attraction of $\theta_{1}$ and $\theta_{2}$ for different damping coefficients. Furthermore, Figure 9b describes the limit cycles of $\theta_{1}$ and ${\dot{\theta }}_{1}$ for different damping coefficients. It is found that its amplitude decreases with the increase of the damping coefficient, which is consistent with the physical intuition [70]. As the $\bar{\beta }$ increases, the greater the energy consumed and the smaller the amplitude of the oscillation.

### 4.7. Effect of the Gravitational Acceleration on the In-Phase Mode

**Figure 10:**
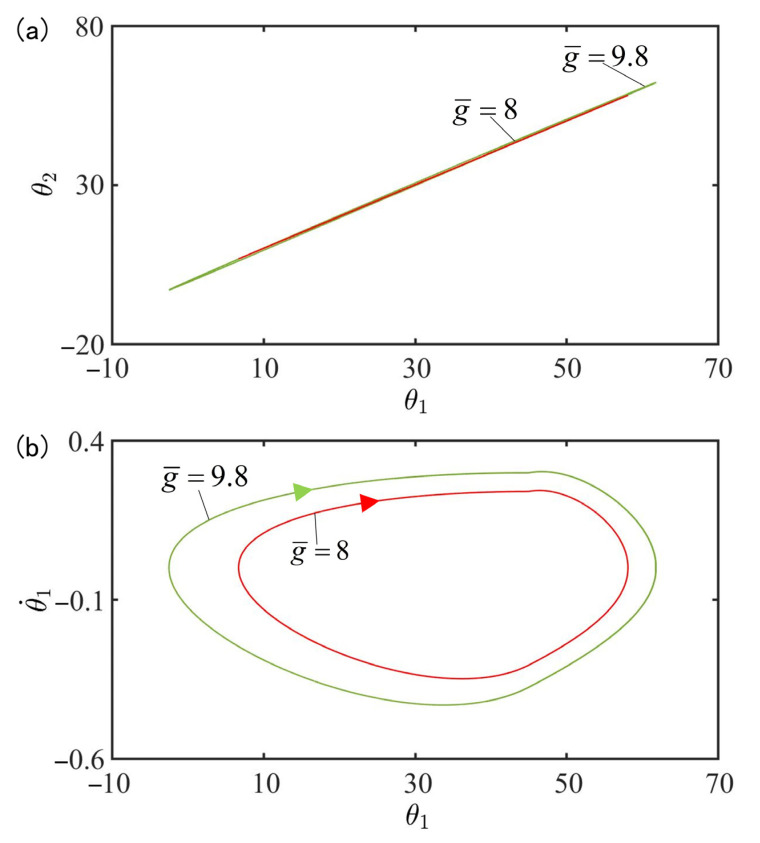
Effect of gravitational acceleration $\bar{g}$ on the in-phase mode for $\bar{I}=0.33$, $C_{0}=0.4$, $\bar{\beta }=0.9$, k=9.2, $\bar{\alpha }=0.1$, ${\dot{\theta }}_{1}^{0}=-0.33$, ${\dot{\theta }}_{2}^{0}=0.33$, $\theta_{u}=45{}^{\circ}$, $\theta_{d}=-80{}^{\circ}$, $\theta_{1}^{0}=0{}^{\circ}$, and $\theta_{2}^{0}=10{}^{\circ}$. (**a**) Domain of attraction of $\theta_{1}$ and $\theta_{2}$ for different gravitational accelerations; (**b**) Limit cycles of $\theta_{1}$ and ${\dot{\theta }}_{1}$ for different gravitational accelerations.

Figure 10 plots the effect of gravitational acceleration $\bar{g}$ on the in-phase mode for $\bar{I}=0.33$, $C_{0}=0.4$, $\bar{\beta }=0.9$, k=9.2, $\bar{\alpha }=0.1$, ${\dot{\theta }}_{1}^{0}=-0.33$, ${\dot{\theta }}_{2}^{0}=0.33$, $\theta_{u}=45{}^{\circ}$, $\theta_{d}=-80{}^{\circ}$, $\theta_{1}^{0}=0{}^{\circ}$, and $\theta_{2}^{0}=10{}^{\circ}$. Figure 10a plots the domains of attraction of $\theta_{1}$ and $\theta_{2}$ for different gravitational accelerations. Furthermore, Figure 10b describes the limit cycles of $\theta_{1}$ and ${\dot{\theta }}_{1}$ for different gravitational accelerations. The results show that the amplitude increases with the increase of gravitational acceleration. According to the physical meaning of $\bar{g}={\left(T_{0}/\sqrt{l_{0}/g}\right)}^{2}$, the larger $\bar{g}$ is, the slower the cis-to-trans conversion is and therefore, the smaller the total moment $M_{D_{1}}$, $M_{D_{2}}$ is, as shown in Equation (7), and the larger the amplitude are.

## 5. Anti-Phase Synchronization Mode

In this section, we further study the mechanism of self-oscillation in anti-phase mode and the influences of dimensionless parameters on the anti-phase synchronous mode in details.

### 5.1. Mechanisms of the Self-Excited Oscillation in Anti-Phase Mode

**Figure 11:**
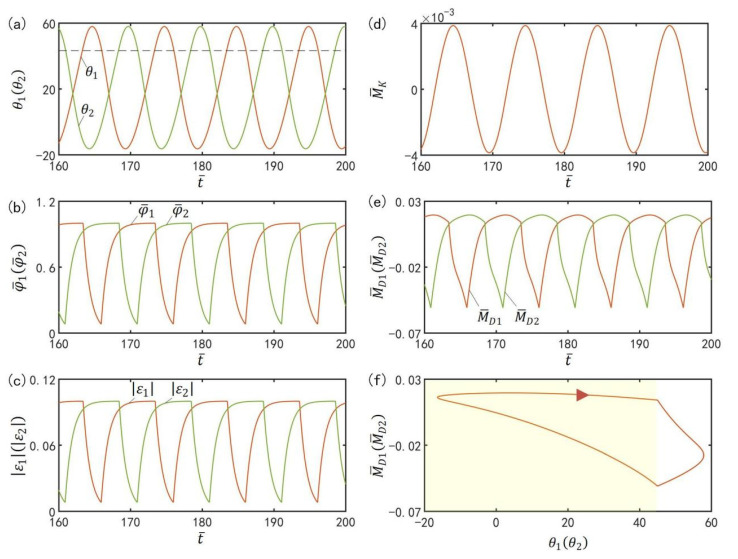
Mechanism of the self-excited oscillation in anti-phase mode. The parameters are $\bar{I}=0.33$, $C_{0}=0.4$, $\bar{g}=9.8$, k=9.2, $\bar{\beta }=0.9$, $\bar{\alpha }=0.03$, ${\dot{\theta }}_{1}^{0}=-0.33$, ${\dot{\theta }}_{2}^{0}=0.33$, $\theta_{u}=45{}^{\circ}$, $\theta_{d}=-80{}^{\circ}$, $\theta_{1}^{0}=0{}^{\circ}$, and $\theta_{2}^{0}=10{}^{\circ}$. (**a**) Time histories of $\theta_{1}$ or $\theta_{2}$. (**b**) Time histories of the number fractions of *cis*-isomers in the two LCE bars. (**c**) Time histories of contraction strains. (**d**) Time history of moment of the torsion spring. (**e**) Time histories of the driving moments. (**f**) The relationship between the driving moments and $\theta_{1}$ or $\theta_{2}$.

To investigate the mechanism of the self-excited oscillation in anti-phase mode of the two LCE oscillators under steady illumination, Figure 11 presents time histories of some key physical quantities of the anti-phase mode in Figure 2a,c. Figure 11a plots the time histories of $\theta_{1}$ and $\theta_{2}$, which show that the two LCE bars oscillate periodically in anti-phase mode. Figure 11b plots time histories of the number fractions of *cis*-isomers in the two LCE bars. Similarly, the number fractions of *cis*-isomers also increase in the light zone while decreasing in the dark zone. Therefore, the contraction strains in the two LCE bars increase in the light zone while decreasing in the dark zone, as shown in Figure 11c. In Figure 11d, the moment of the torsion spring on the two bars changes periodically, because the angle difference between the two bars in anti-phase mode varies periodically. In Figure 11e, the driving moments of the two oscillators in anti-phase mode also fluctuates periodically. Figure 11f delineates the dependence of the driving moment on $\theta_{1}$ or $\theta_{2}$. In Figure 11f, the area surrounded by the closed curve represents the net work performed by the steady illumination during one cycle of the self-excited oscillation, which compensates for the energy loss caused by damping to maintain the oscillation of the LCE oscillators.

### 5.2. Effect of the Spring Constant on the Anti-Phase Mode

**Figure 12:**
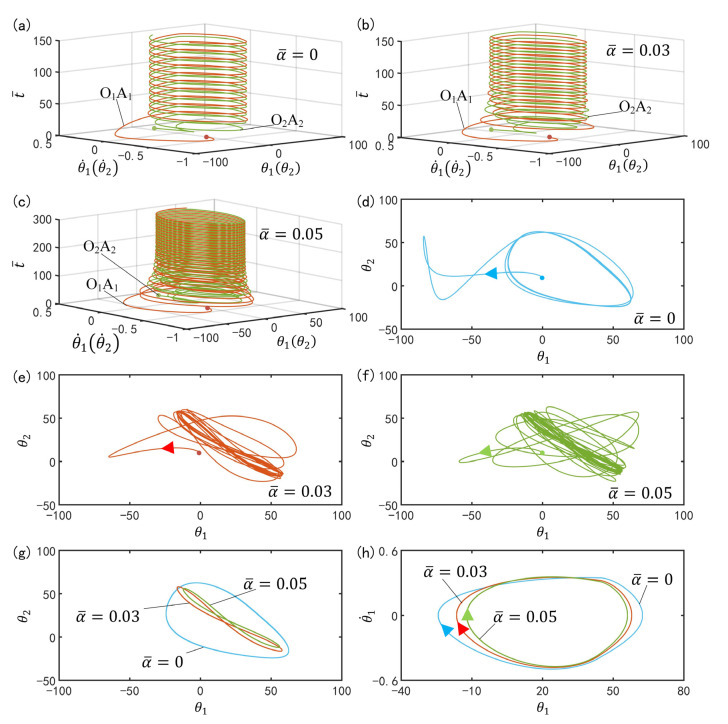
Effect of spring constant on the anti-phase mode. The parameters are $\bar{I}=0.33$, $C_{0}=0.4$, $\bar{g}=9.8$, k=9.2, $\bar{\beta }=0.9$, ${\dot{\theta }}_{1}^{0}=-0.33$, ${\dot{\theta }}_{2}^{0}=0.33$, $\theta_{u}=45{}^{\circ}$, $\theta_{d}=-80{}^{\circ}$, $\theta_{1}^{0}=0{}^{\circ}$, and $\theta_{2}^{0}=10{}^{\circ}$. (**a**) Time history for $\bar{\alpha }=0$; (**b**) Time history for $\bar{\alpha }=0.03$; (**c**) Time history for $\bar{\alpha }=0.05$; (**d**) Phase diagrams for $\bar{\alpha }=0$; (**e**) Phase diagrams for $\bar{\alpha }=0.03$; (**f**) Phase diagrams for $\bar{\alpha }=0.05$; (**g**) Domain of attraction of $\theta_{1}$ and $\theta_{2}$ for different spring constants; (**h**) Limit cycles of $\theta_{1}$ and ${\dot{\theta }}_{1}$ for different spring constants.

Figure 12a–c plot the time history for different spring constants. In the computation, we set $\bar{I}=0.33$, $C_{0}=0.4$, $\bar{g}=9.8$, k=9.2, $\bar{\beta }=0.9$, ${\dot{\theta }}_{1}^{0}=-0.33$, ${\dot{\theta }}_{2}^{0}=0.33$, $\theta_{u}=45{}^{\circ}$, $\theta_{d}=-80{}^{\circ}$, $\theta_{1}^{0}=0{}^{\circ}$, and $\theta_{2}^{0}=10{}^{\circ}$. For $\bar{\alpha }=0$, the phase difference between the two bars after a period of time is a constant value that is generally not 180° or 0°, as shown in Figure 12a. Figure 12d plots the phase diagrams for $\bar{\alpha }=0$, in which $\theta_{1}$ and $\theta_{2}$ evolve from the initial disorder into a final attraction domain. The domains of attraction of $\theta_{1}$ and $\theta_{2}$ and limit cycles of $\theta_{1}$ and ${\dot{\theta }}_{1}$ are further plotted in Figure 12g,h, respectively. Through calculation, we find that for $\bar{\alpha }=0$, the final phase difference of two bars depends on initial conditions, while the limit cycles are independent on the initial conditions. It can be understood that for $\bar{\alpha }=0$, the system is equivalent to the single oscillator [66], and the phase difference is determined by their independent self-excited oscillations.

In Figure 12b,c, for $\bar{\alpha }=0.03$ and $\bar{\alpha }=0.05$, the self-excited oscillations of the two LCE oscillators have a phase difference of half a cycle after a period of time, which means that the two bars oscillate synchronously in anti-phase mode. Figure 11e,f plot the phase diagrams for $\bar{\alpha }=0.03$ and $\bar{\alpha }=0.05$, in which $\theta_{1}$ and $\theta_{2}$ evolve from the initial disorder into a final attraction domain. Furthermore, Figure 12g,h plot the domains of attraction of $\theta_{1}$ and $\theta_{2}$ and the limit cycles of $\theta_{1}$ and ${\dot{\theta }}_{1}$. Obviously, the domains of attraction and the limit cycles are also different. It can be seen that its amplitude decreases with the increase of the spring constant. Careful calculation shows that the period also decreases with the increase of the spring constant. In fact, the system in anti-phase mode for $\bar{\alpha }<{\bar{\alpha }}_{\mathrm{crit}}$ is equivalent to a single self-excited oscillator constrained by a fixed torsion spring with half the original length, as shown in Figure 5b. The greater the spring constant, the smaller the amplitude and the period. This result is consistent with the physical intuition [70].

### 5.3. Effect of Initial Conditions on the Anti-Phase Mode

**Figure 13:**
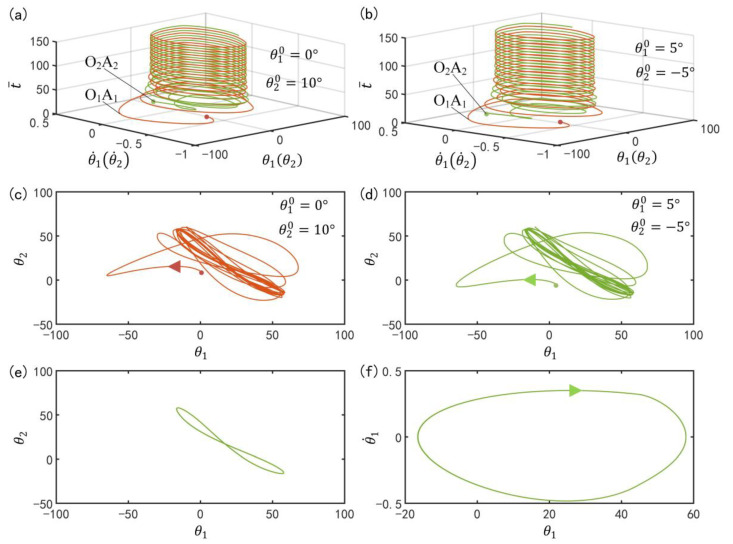
Effect of initial conditions on the anti-phase mode. The parameters are $\bar{I}=0.33$, $C_{0}=0.4$, $\bar{g}=9.8$, k=9.2, $\bar{\beta }=0.9$, $\bar{\alpha }=0.03$, ${\dot{\theta }}_{1}^{0}=-0.33$, ${\dot{\theta }}_{2}^{0}=0.33$, $\theta_{u}=45{}^{\circ}$, and $\theta_{d}=-80{}^{\circ}$. (**a**) Time history for $\theta_{1}^{0}=0{}^{\circ}$ and $\theta_{2}^{0}=10{}^{\circ}$; (**b**) Time history for $\theta_{1}^{0}=5{}^{\circ}$ and $\theta_{2}^{0}=-5{}^{\circ}$; (**c**) Phase diagrams for $\theta_{1}^{0}=0{}^{\circ}$ and $\theta_{2}^{0}=10{}^{\circ}$; (**d**) Phase diagrams for $\theta_{1}^{0}=5{}^{\circ}$ and $\theta_{2}^{0}=-5{}^{\circ}$; (**e**) Domain of attraction of $\theta_{1}$ and $\theta_{2}$ for different initial conditions; (**f**) Limit cycles of $\theta_{1}$ and ${\dot{\theta }}_{1}$ for different initial conditions.

Figure 13a,b plot the time histories for two different initial conditions. In the computation, we set $\bar{I}=0.33$, $C_{0}=0.4$, $\bar{g}=9.8$, k=9.2, $\bar{\beta }=0.9$, $\bar{\alpha }=0.03$, ${\dot{\theta }}_{1}^{0}=-0.33$, ${\dot{\theta }}_{2}^{0}=0.33$, $\theta_{u}=45{}^{\circ}$, and $\theta_{d}=-80{}^{\circ}$. In Figure 13a,b, for two different initial conditions, the self-excited oscillations of the two LCE oscillators have a phase difference of half a cycle after a period of time, which means that the two bars oscillate synchronously in anti-phase mode. Figure 13c,d plot the phase diagrams for the two initial conditions, in which $\theta_{1}$ and $\theta_{2}$ evolve from the initial disorder into a final attraction domain. Figure 13e further plots the domains of attraction of $\theta_{1}$ and $\theta_{2}$ for different initial conditions. The calculation shows that the two domains of attraction are the same. The results also show that the initial condition has no effect on the synchronous mode.

Furthermore, Figure 13f describes the limit cycles of $\theta_{1}$ and ${\dot{\theta }}_{1}$ for different initial conditions. Similarly, the two limit cycles are also identical. This implies that the amplitude and frequency of the LCE oscillators are independent on the initial condition. More calculations also show that the influence of initial conditions is the same. It is noted that the effect of initial condition on amplitude and frequency of the anti-phase mode is similar to that of the single LCE oscillator [66].

### 5.4. Effect of the Contraction Coefficient on the Anti-Phase Mode

**Figure 14:**
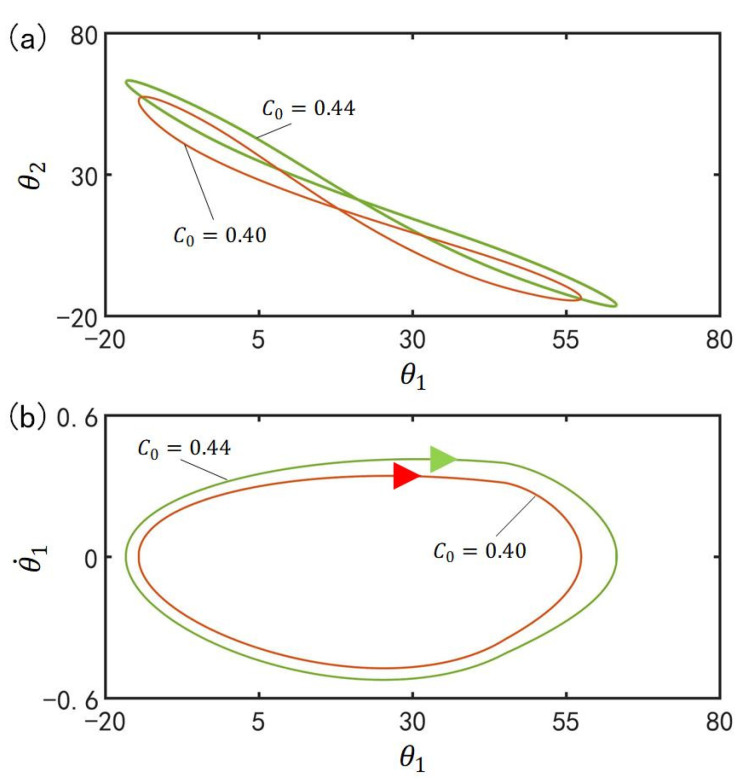
Effect of contraction coefficient $C_{0}$ on the anti-phase mode for $\bar{I}=0.33$, $\bar{\alpha }=0.03$, $\bar{g}=9.8$, k=9.2, $\bar{\beta }=0.9$, ${\dot{\theta }}_{1}^{0}=-0.33$, ${\dot{\theta }}_{2}^{0}=0.33$, $\theta_{u}=45{}^{\circ}$, $\theta_{d}=-80{}^{\circ}$, $\theta_{1}^{0}=0{}^{\circ}$, and $\theta_{2}^{0}=10{}^{\circ}$. (**a**) Domain of attraction of $\theta_{1}$ and $\theta_{2}$ for different contraction coefficients; (**b**) Limit cycles of $\theta_{1}$ and ${\dot{\theta }}_{1}$ for different contraction coefficients.

Figure 14 plots the effect of contraction coefficient $C_{0}$ on the anti-phase mode for $\bar{I}=0.33$, $\bar{\alpha }=0.03$, $\bar{g}=9.8$, k=9.2, $\bar{\beta }=0.9$, ${\dot{\theta }}_{1}^{0}=-0.33$, ${\dot{\theta }}_{2}^{0}=0.33$, $\theta_{u}=45{}^{\circ}$, $\theta_{d}=-80{}^{\circ}$, $\theta_{1}^{0}=0{}^{\circ}$ and $\theta_{2}^{0}=10{}^{\circ}$. Figure 14a plots the domains of attraction of $\theta_{1}$ and $\theta_{2}$ for different contraction coefficients. Furthermore, Figure 14b describes the limit cycles of $\theta_{1}$ and ${\dot{\theta }}_{1}$ for different contraction coefficients. Similar to the effect of contraction coefficient on the in-phase mode, the amplitude in anti-phase mode increases with the increase of the contraction coefficient. With the rise of contraction coefficient $C_{0}$, the strain of the LCE bar increases and then the center of gravity of the LCE bar changes greatly. Therefore, the driving moment increases and the amplitude of the self-excited oscillation ascends.

### 5.5. Effect of the Light Intensity on the Anti-Phase Mode

**Figure 15:**
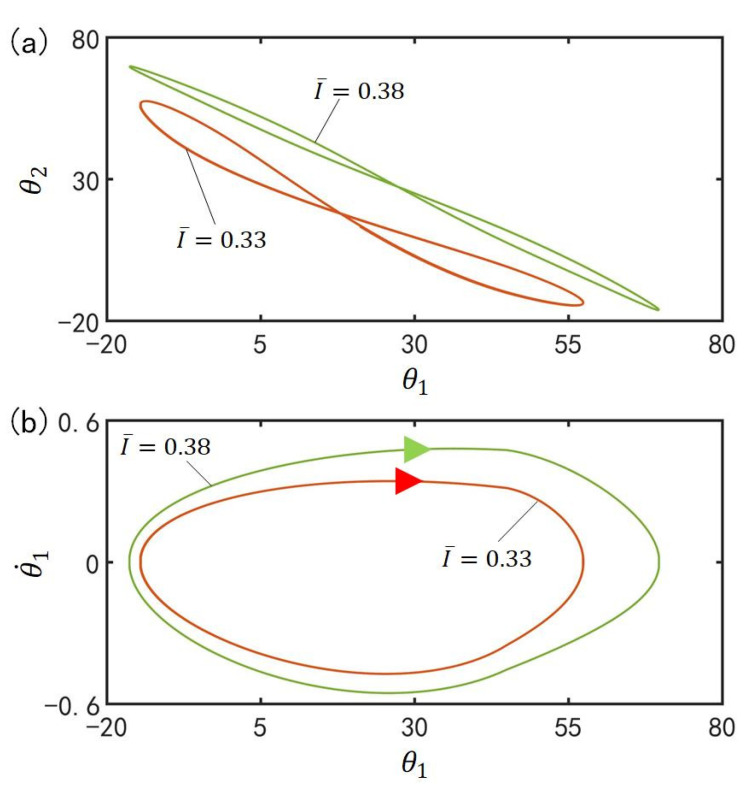
Effect of light intensity $\bar{I}$ on the anti-phase mode for $C_{0}=0.4$, $\bar{\alpha }=0.03$, $\bar{g}=9.8$, k=9.2, $\bar{\beta }=0.9$, ${\dot{\theta }}_{1}^{0}=-0.33$, ${\dot{\theta }}_{2}^{0}=0.33$, $\theta_{u}=45{}^{\circ}$, $\theta_{d}=-80{}^{\circ}$, $\theta_{1}^{0}=0{}^{\circ}$, and $\theta_{2}^{0}=10{}^{\circ}$. (**a**) Domain of attraction of $\theta_{1}$ and $\theta_{2}$ for different light intensities; (**b**) Limit cycles of $\theta_{1}$ and ${\dot{\theta }}_{1}$ for different light intensities.

Figure 15 plots the effect of light intensity $\bar{I}$ on the anti-phase mode for $C_{0}=0.4$, $\bar{\alpha }=0.03$, $\bar{g}=9.8$, k=9.2, $\bar{\beta }=0.9$, ${\dot{\theta }}_{1}^{0}=-0.33$, ${\dot{\theta }}_{2}^{0}=0.33$, $\theta_{u}=45{}^{\circ}$, $\theta_{d}=-80{}^{\circ}$, $\theta_{1}^{0}=0{}^{\circ}$, and $\theta_{2}^{0}=10{}^{\circ}$. Figure 15a plots the domains of attraction of $\theta_{1}$ and $\theta_{2}$ for different light intensities. Furthermore, Figure 15b describes the limit cycles of $\theta_{1}$ and ${\dot{\theta }}_{1}$ for different light intensities. Its amplitude also increases with the augmentation of light intensity. The rise of light intensity $\bar{I}$ magnifies the light-driven contraction of the LCE bar, and then the center of gravity of the LCE bar changes greatly. Eventually, the driving moment and the amplitude of the self-excited oscillation are augmented.

### 5.6. Effect of the Damping Coefficient on the Anti-Phase Mode

**Figure 16:**
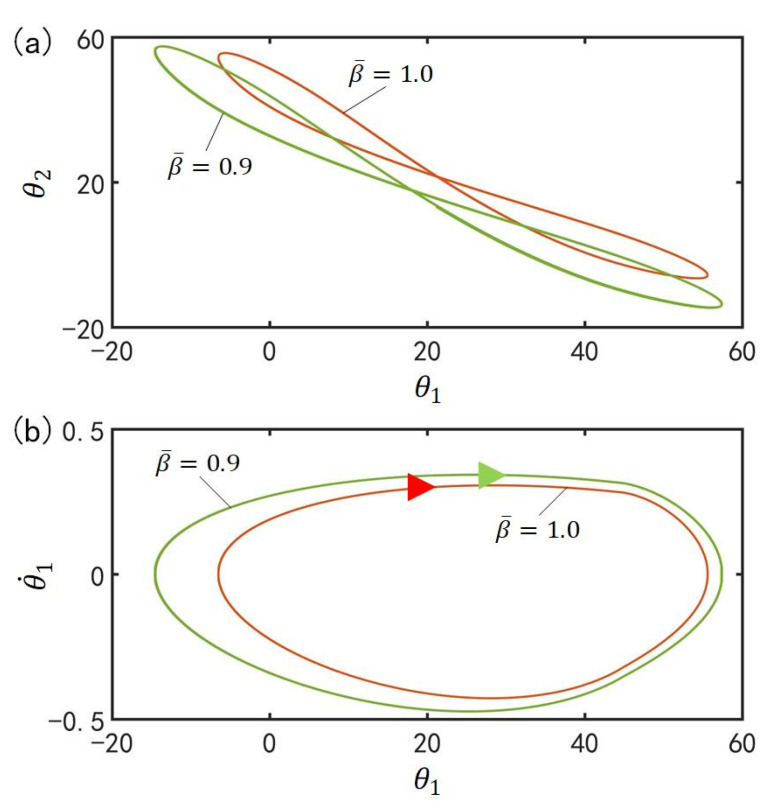
Effect of damping coefficient $\bar{\beta }$ on the anti-phase mode for $\bar{I}=0.33$, $C_{0}=0.4$, $\bar{g}=9.8$, k=9.2, $\bar{\alpha }=0.03$, ${\dot{\theta }}_{1}^{0}=-0.33$, ${\dot{\theta }}_{2}^{0}=0.33$, $\theta_{u}=45{}^{\circ}$, $\theta_{d}=-80{}^{\circ}$, $\theta_{1}^{0}=0{}^{\circ}$, and $\theta_{2}^{0}=10{}^{\circ}$. (**a**) Domain of attraction of $\theta_{1}$ and $\theta_{2}$ for different damping coefficients; (**b**) Limit cycles of $\theta_{1}$ and ${\dot{\theta }}_{1}$ for different damping coefficients.

Figure 16 plots the effect of damping coefficient $\bar{\beta }$ on the anti-phase mode for $\bar{I}=0.33$, $C_{0}=0.4$, $\bar{g}=9.8$, k=9.2, $\bar{\alpha }=0.03$, ${\dot{\theta }}_{1}^{0}=-0.33$, ${\dot{\theta }}_{2}^{0}=0.33$, $\theta_{u}=45{}^{\circ}$, $\theta_{d}=-80{}^{\circ}$, $\theta_{1}^{0}=0{}^{\circ}$, and $\theta_{2}^{0}=10{}^{\circ}$. Figure 16a plots the domains of attraction of $\theta_{1}$ and $\theta_{2}$ for different damping coefficients. Furthermore, Figure 16b describes the limit cycles of $\theta_{1}$ and ${\dot{\theta }}_{1}$ for different damping coefficients. Similarly, its amplitude also decreases with the rise of the damping coefficient. This result can be understood as follows: the more $\bar{\beta }$ increases, the greater the energy consumed and the smaller the amplitude.

### 5.7. Effect of the Gravitational Acceleration on the Anti-Phase Mode

**Figure 17:**
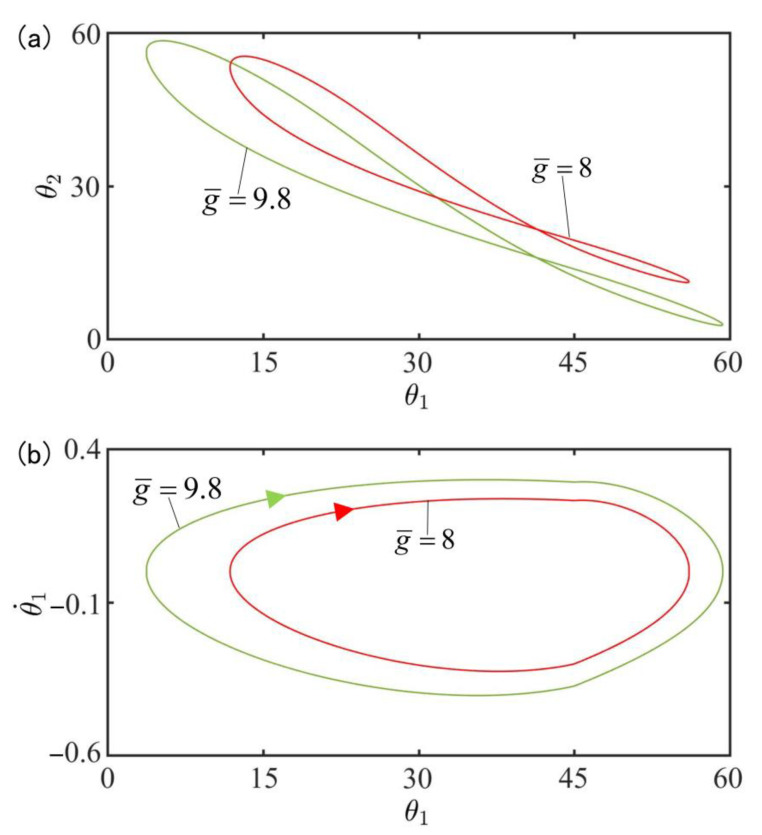
Effect of gravitational acceleration $\bar{g}$ on the anti-phase mode for $\bar{I}=0.33$, $C_{0}=0.4$, $\bar{\beta }=0.9$, k=9.2, $\bar{\alpha }=0.03$, ${\dot{\theta }}_{1}^{0}=-0.33$, ${\dot{\theta }}_{2}^{0}=0.33$, $\theta_{u}=45{}^{\circ}$, $\theta_{d}=-80{}^{\circ}$, $\theta_{1}^{0}=0{}^{\circ}$, and $\theta_{2}^{0}=10{}^{\circ}$. (**a**) Domain of attraction of $\theta_{1}$ and $\theta_{2}$ for different gravitational accelerations; (**b**) Limit cycles of $\theta_{1}$ and ${\dot{\theta }}_{1}$ for different gravitational accelerations.

Figure 17 plots the effect of gravitational acceleration $\bar{g}$ on the anti-phase mode for $\bar{I}=0.33$, $C_{0}=0.4$, $\bar{\beta }=0.9$, k=9.2, $\bar{\alpha }=0.03$, ${\dot{\theta }}_{1}^{0}=-0.33$, ${\dot{\theta }}_{2}^{0}=0.33$, $\theta_{u}=45{}^{\circ}$, $\theta_{d}=-80{}^{\circ}$, $\theta_{1}^{0}=0{}^{\circ}$ and $\theta_{2}^{0}=10{}^{\circ}$. Figure 17a plots the domains of attraction of $\theta_{1}$ and $\theta_{2}$ for different gravitational accelerations. Furthermore, Figure 17b describes the limit cycles of $\theta_{1}$ and ${\dot{\theta }}_{1}$ for different gravitational accelerations. Similarly, its amplitude also increases with the rise of the gravitational acceleration. This result can be understood as follows: the more $\bar{g}$ increases, the slower the cis-to-trans conversion, the smaller the total moment $M_{D_{1}}$, $M_{D_{2}}$ in Equation (7), and thus the smaller the amplitude.

## 6. Conclusions

The synchronization and group behaviors of self-excited coupled oscillators are common in nature and deserve to be explored, for self-excited motions have the advantages of actively collecting energy from the environment, being autonomous, making equipment portable, and so on. Based on self-excited oscillators composed of LCE bars, the synchronization of two identical self-excited oscillators is theoretically investigated in this paper. By combing the well-established dynamic LCE model, a theoretical model for the self-excited motion of the two self-excited oscillators connected by a torsion spring and synchronization of the self-excited motion is numerically calculated by MATLAB software. It is found that self-excited oscillation of the system has two synchronization modes: in-phase mode and anti-phase mode. By plotting the time histories of various quantities, we elucidate the mechanism of self-excited oscillation and the two synchronization modes.

The numerical calculations show that the synchronization mode is mainly determined by the interaction between the two identical self-excited oscillators. For strong interactions with the spring constant, the system always develops into in-phase synchronization mode, and both its amplitude and period are independent on the spring constant. In this case, the system is equivalent to two identical self-excited oscillators without interaction. For weak interaction with a small spring constant, the system will evolve into anti-phase synchronization mode, and its amplitude decreases with the increase of the interaction. In this case, it is equivalent to single self-excited oscillator constrained by fixed torsion spring with half original length.

Furthermore, the effects of initial conditions, contraction coefficient, light intensity and damping coefficient on the two synchronization modes of the self-excited oscillation are investigated systematically, by plotting their domain of attraction of the system. The initial condition generally does not affect the synchronization mode or its amplitude. The amplitude of the self-oscillation always increases with the increasing contraction coefficient, gravitational acceleration, and light intensity, while decreasing with the increasing damping coefficient.

In the future, the proposed design in this work is worth experimental implementation to validate the predicted phenomena. It is recommended that the LCE sample with a large contraction coefficient and small thickness is fabricated and a high light intensity is used in experiments. This study will deepen people’s understanding of the synchronization of self-excited oscillators and provide promising applications in energy acquisition, power generation, monitoring, soft robotics, medical equipment, and micro/nanodevices. Meanwhile, the theoretical framework could be extended to scenarios involving large-scale synchronization of systems with numerous interacting oscillators and suggests possible avenues for future research in this direction.

## Figures and Tables

**Table 1:** Material properties and geometric parameters.

Parameter	Definition	Value	Units
$C_{0}$	Contraction coefficien	0~0.4	/
$T_{0}$	Trans-to-cis thermal relaxation time	1~100	ms
$I_{0}$	Light intensity	0~11	kW/m^2^
$\eta_{0}$	Light-absorption constant	0.0003	m^2^/(s·W)
α	Spring constant	0~0.0011	N/m
β	Damping coefficient	0~0.5	kg/s
$l_{0}$	Length of LCE bar	10	mm
g	Gravitational acceleration	9.8	m/s^2^
m	Mass of LCE bar	10	g

**Table 2:** Dimensionless parameters.

Parameter	$\bar{I}$	$\bar{g}$	$\bar{\alpha }$	$\bar{\beta }$	$C_{0}$
Value	0~0.33	0.001~10	0~0.11	0~1	0~0.4

## Data Availability

Not applicable.
